# Clinical evaluation for the difference of absorbed doses calculated to medium and calculated to water by Monte Carlo method

**DOI:** 10.1186/s13014-018-1081-3

**Published:** 2018-07-28

**Authors:** Li Chen, Botian Huang, Xiaoyan Huang, Wufei Cao, Wenzhao Sun, Xiaowu Deng

**Affiliations:** 1 Department of Radiation Oncology, Sun Yat-Sen University Cancer Center, State Key Laboratory of Oncology in South China, Collaborative Innovation Center for Cancer Medicine, Guangdong Key Laboratory of Nasopharyngeal Carcinoma Diagnosis and Therapy, No. 651, Dongfeng Road East, Guangzhou, 510060 China; 2 Department of Radiation Oncology, The first Affiliated Hospital of Sun Yat-Sen University, Guangzhou, 510060 China

**Keywords:** Monte Carlo dose calculation, Absorbed dose to medium, Absorbed dose to water, Nasopharyngeal cancer, Gamma analysis

## Abstract

**Background:**

To evaluate the difference of absorbed doses calculated to medium and to water by a Monte Carlo (MC) algorithm based treatment planning system (TPS), and to assess the potential clinical impact to dose prescription.

**Methods:**

Thirty patients, 10 nasopharyngeal cancer (NPC), 10 lung cancer and 10 bone metastases cases, were selected for this study. For each case, the treatment plan was generated using a commercial MC based TPS and dose was calculated to medium (D_m_). The plan was recalculated for dose to water (D_w_) using the same Monitor Units (MU) and control points. The differences between D_m_ and D_w_ were qualitatively evaluated by dose-volume parameters and by the plan subtraction method. All plans were measured using the MapCheck2, and gamma passing rates were calculated.

**Results:**

For NPC and Lung cases, the mean differences between D_w_ and D_m_ for the targets were less than 2% and the maximum difference was 3.9%. The maximum difference of D_2%_ for the organs at risk (OARs) was 6.7%. The maximum differences between D_w_ and D_m_ were as high as 10% in certain high density regions. For bone metastases cases, the mean differences between D_w_ and D_m_ for the targets were more than 2.2% and the maximum difference was 7.1%. The differences between D_w_ and D_m_ for the OARs were basically negligible. At 3%&3 mm criterion, the gamma passing rate of D_w_ plan and D_m_ plan were close (> 94%).

**Conclusion:**

The differences between D_w_ and D_m_ has little clinical impact for most clinical cases. In bony structures the differences may become clinically significant if the target/OAR is receiving doses close to its tolerance limit which can potentially influence the selection or rejection of a particular plan.

## Background

Absorbed dose is an important parameter in characterizing the effect of radiation therapy for the efficacy of tumor eradication and protection from unacceptable damage to normal organs [1]. For historical reasons, in terms of dose, D_w_ has been assumed for reporting the dose to various media. However, human body is not only composed of water. Many tissues in the body have different densities than water, especially the bones and lung. For radiation therapy the dose absorbed to water cannot accurately represent the actual dose absorbed in different tissues. In practice, traditional treatment planning system (TPS) typically takes the effect of different tissue densities with attenuation and scatter into considerations but reports the dose at each location as the dose to water. Monte Carlo (MC) algorithm is the most accurate algorithm for dose calculation in that it simulates the transport properties of various particles in various media in the region of interest and scores the dose contribution locally to the medium with its assigned chemical composition as well as density. The resulting dose distributions may be different from those calculated by traditional dose calculation algorithms, especially for tissues of heterogeneity [2–4]. In recent years, MC has been increasingly adopted in clinical application [5–7]. There are a number of reasons for using D_w_ for reporting of MC calculated doses. Two major ones are that it has been used in decades of clinical studies for outcome correlation with the dose, and that the calibration protocols are all referenced to water. A technical issue related to dose calibration is that an MC based TPS could model the chemical composition of various biological tissues by approximation as a function of Computed Tomography (CT) numbers based on data of the human body (reference International Commission on Radiation Units & Measurements reports 44 and 46). Such an approximation may not perform well for non-biological materials like in a quality assurance (QA) phantom. MC based dose calculations typically report absorbed dose to media (D_m_). Therefore there is a need to convert between D_m_ and D_w_, and, as Siebers JV et al. [8] argued, MC is capable of doing the conversion. Siebers et al. presented a method to calculate the difference between D_m_ and D_w_ by applying the Bragg-Gray cavity theory, and their results showed a difference exceeding 10% in cortical bones.

Currently there is no consensus regarding whether D_m_ or D_w_ should be used for an MC based TPS [9, 10]. When it comes to clinical application, the difference between D_w_ and D_m_ will affect interpretation of dose distribution and perhaps the value of prescription dose, leading to differences in plan evaluation, dose reporting, and dose verification. In this work, D_m_ and D_w_ were both calculated using Monaco TPS for 10 nasopharyngeal cancer (NPC) cases, 10 lung cancer cases and 10 bone target cases, in order to investigate the issue in two common clinical sites in which differences of dose distributions may be highlighted. Dose Volume Histogram (DVH) was used to analyze dose parameters in the target and organ at risk (OAR), and three dimensional dose difference distributions between D_m_ and D_w_ were calculated. Gamma passing rates (measurement results vs D_m_/D_w_ plans) were calculated at different QA criteria to evaluate the dose accuracy.

## Methods

### D_m_ plan originally created for treatment

Ten NPC cases in stage T3 or T4, 10 lung cancer cases and 10 bone target cases (7 cases of lumbar vertebra metastasis, 3 cases of thoracic vertebra metastasis) treated at Sun Yat-sen University Cancer Center were retrospectively chosen in this study. The gross tumor volumes (GTVs) and clinical tumor volume (CTV) were contoured by experienced radiation oncologists according to definitions in the ICRU 50 and ICRU 62 reports [11, 12], and the planning target volume (PTV) were generated following a set of physician prescribed margins that were consistent with departmental protocols specific to the disease sites. Monaco TPS (Version 5.0, Elekta) was used to create the treatment plans for step-and-shoot IMRT with an Elekta Synergy linac, and MC calculated D_m_ was chosen for dose reporting. Nine equally spaced fields were used for NPC cases. The prescription of NPC cases and Lung cancer cases were 70 Gy (32 or 33 fractions, 5 days/week) and 65 Gy (26 fractions, 5 days/week) respectively. The main planning objectives for NPC are PTV V_100%_ > 98% and PTV V_110%_ < 10% (V_x%_, is the percentage volume of reign of interest (ROI) that receives at least x% prescription dose), spinal cord D_2%_ < 45Gy, brain stem D_2%_ < 54Gy, parotid gland D_50%_ < 30Gy, optical nerve D_2%_ < 54Gy, and the dose to lens as low as possible. For lung IMRT cases 5–7 fields were used. The planning objectives are PTV V_100%_ > 95% and PTV V_110%_ < 2%, spinal cord D_2%_ < 45Gy, normal lung V_20 Gy_ < 35% (V_D Gy_, is the percentage volume of ROI that receives at least absorbed dose D) and normal lung mean dose <19Gy, heart V_30 Gy_ < 40%, and the maximum esophagus dose <65Gy. For bone target cases, 5–7 fields were used. The prescription of bone target cases was 25 Gy (5Gy/fractions, 5 days/week). The main planning objectives are for PTV, V_100%_ > 95% and V_110%_ < 10%, for spinal cord D_max_ < 26 Gy, for lung V_10Gy_ < 15%, and the maximum esophagus dose < 26 Gy.

### D_w_ calculation

The MC algorithm in the Monaco TPS used for this study, called XVMC, calculates dose based upon mass density. A technical issue of dose calculation with MC in treatment planning is how to obtain the density and chemical composition data for the patient model from the CT. An approximation is made by assigning a voxel to certain type of tissue in the human body based on its Hounsfield unit (HU) in a certain range, and the mass density and composition data can be looked up in the International Commission on Radiation Units & Measurements Reports No. 46 [13]. XVMC algorithm converts CT numbers to ED numbers using the user-defined CT-to-ED calibration table and takes with a fit function that maps continuously the electron density to mass density for matching a tissue with approximating cross section and attenuation coefficient data [14].

The conversion to D_w_ can be calculated based on the distribution of D_m_ plan according to the Bragg-Gray cavity theory:

1 $$ {\mathrm{D}}_{\mathrm{w}}={\mathrm{D}}_{\mathrm{m}}\ {s}_{w, med} $$

where *s*_*w,med*_ is the mean unconstrained mass stop power ratio of water to media of primary electron spectrum, and D_w_ is understood as the dose to the voxel replacement of water embedded to the actual media. Theoretically mass stop power ratio can be calculated by the following formula [8]:

2 $$ {s}_{w, med}={\int}_0^{E_{max}}{\left({\Phi}_E\right)}_m{\left(S/\rho \right)}_w dE/{\int}_0^{E_{max}}{\left({\Phi}_E\right)}_m{\left(S/\rho \right)}_{med} dE $$

where (*S*/*ρ*)_*w*_ and (*S*/*ρ*)_*med*_ are the unconstrained mass stop power of water and media, respectively. (Φ_*E*_)_*m*_ is the primary electron fluence in the medium and *E*_*max*_ is the maximum energy in the (Φ_*E*_)_*m*_ distribution. The stopping power ratio in Moncao was pre-calculated by approximation for tissue-like media.

The conversion from D_m_ to D_w_ in Monaco with a clinically accepted plan involved a simple recalculation with exactly the same set of plan parameters (all the geometric parameters and monitor units (MU)) retained. The stopping power ratios dependent of mass density were applied voxel by voxel. The matrix of dose calculation grid was 0.3 cm × 0.3 cm × 0.3 cm, and the Monte Carlo statistical uncertainty was set at 3% per control point.

### D_m_ and D_w_ dose verification

All the plans were measured with MapCHECK2 (Sun Nuclear, Florida, USA) to verify the dose distribution. MacpCHECK2 was mounted in a water-equivalent phantom (MapPHAN) with a 5 cm equivalent depth from the surface to the detectors. The TPS planed dose was calculated on the real phantom CT images without overriding the density. The measured dose distributions of composite fields were compared with the corresponding planned dose distributions (D_m_ or D_w_), and the local dose normalization gamma (*γ*) passing rates were calculated at the setting dose difference (DD) and distance to agreement (DTA). In order to eliminate dose in the out-of-field region where a large relative dose difference can be calculated and hence skew the*γ* result, a lower dose threshold (10%) was set and below the threshold the*γ* result was ignored. Using 3%&3 mm, 2%&2 mm and 1%&1 mm tolerances, the gamma passing rates were calculated to find how the pass rates change with reduction of dose difference and DTA limits.

### Data analysis

According to the ICRU 83 report, the volume-dose is recommended to describe the dose information in the ROIs, as D_x%_ to note the dose that X% of volume of ROI receives [15]. For example, D_98%_ means 98% of volume received the dose at specified value such as 65Gy. These DVH parameters were used for statistical analysis of D_w_ and D_m_ dose distributions. The bin width of the DVHs was 1 cGy, and the resolution for DVH sampling was 0.1 cm. The difference between the D_w_ and D_m_ was calculated by:

3 $$ \mathrm{Diff}\ \left(\%\right)=\left({\left({\mathrm{D}}_{\mathrm{x}\%}\right)}_{\mathrm{w}}-{\left({\mathrm{D}}_{\mathrm{x}\%}\right)}_{\mathrm{m}}\right)/{\left({\mathrm{D}}_{\mathrm{x}\%}\right)}_{\mathrm{w}}\times 100 $$

The plan subtraction method was used to evaluate the spatial dose difference distribution of D_w_ and D_m_.

Paired t-tests were performed using the SPSS software (Version 19, SPSS, Inc., USA) to determine the statistical significance of the difference between D_w_ and D_m_, with a *p*-value < 0.05 as the threshold for consideration as statistically significant.

## Results

### D_w_ and D_m_ for NPC cases

**Fig. 1:**
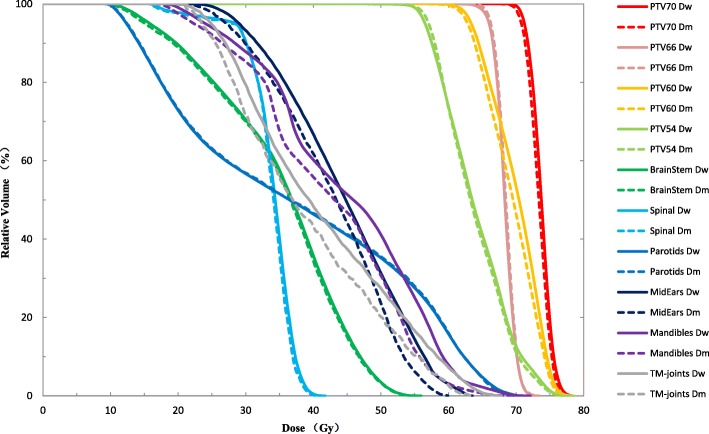
DVH comparison for D_w_ and D_m_ results from the MC-based Monaco TPS for a typical NPC case

Figure 1 shows the comparison of the DVH results with D_w_ and D_m_ for a typical NPC treatment plan. There were small but systematic deviations from D_m_ to D_w_ in the planning target volumes (PTVs). Table 1 shows the mean and difference in dose-volume indices calculated with MC, evaluated for 10 NPC cases. Except for the D_50%_ and D_2%_ of PTV66, and D_98%_ of PTV54, all DVH indices for all PTVs were different with statistical significance (*p* <  0.05), including D_98%_, D_50%_, and D_2%_ (D_x%_, the minimum dose that x% of the volume of the organ receives from the cumulative DVH). The possible reason for PTV66 behaved differently from the others may be that PTV66 is the lymph gland target, small in size and relatively variable in location among different patients. For the D_2%_ of PTV70, PTV66, PTV60 and PTV54, the values of the D_m_ plan are less than that of D_w_, and the mean deviation was 1.9 ± 1.1%, 0.4 ± 1.0%, 1.7 ± 1.0% and 1.3 ± 0.7%, respectively. The difference between D_w_ and D_m_ in the mean dose of PTVs were within 1%.

**Table 1:** The mean and standard deviation of D_w_ and D_m_ in dose-volume indices calculated with Monte Carlo for 10 NPC IMRT cases

ROI	Parameter	D_w_(Gy)	D_m_(Gy)	Diff(%)	*p*
PTV70	D_98%_	70.7 ± 0.6	70.2 ± 0.3	0.7 ± 0.5	0.002
	D_50%_	74.3 ± 0.5	73.6 ± 0.3	0.9 ± 0.4	< 0.001
	D_2%_	78.2 ± 1.5	76.7 ± 1.0	1.9 ± 1.1	0.001
PTV66	D_98%_	64.6 ± 2.1	65.0 ± 2.2	−0.6 ± 0.4	0.002
	D_50%_	69.0 ± 0.6	69.0 ± 0.6	0.1 ± 0.3	0.923
	D_2%_	72.4 ± 1.2	72.1 ± 1.5	0.4 ± 1.0	0.235
PTV60	D_98%_	63.2 ± 1.1	62.7 ± 1.0	0.7 ± 0.5	0.001
	D_50%_	71.7 ± 0.9	71.1 ± 0.9	0.9 ± 0.3	< 0.001
	D_2%_	77.4 ± 1.3	76.1 ± 0.9	1.7 ± 1.0	< 0.001
PTV54	D_98%_	56.4 ± 0.7	56.6 ± 0.5	0.2 ± 0.4	0.144
	D_50%_	65.0 ± 1.2	64.7 ± 1.2	0.6 ± 0.3	< 0.001
	D_2%_	76.1 ± 1.2	75.1 ± 0.9	1.3 ± 0.7	< 0.001
Spinal Cord	D_50%_	34.0 ± 1.6	33.9 ± 1.7	0.5 ± 0.3	< 0.001
	D_2%_	39.6 ± 1.2	39.2 ± 1.2	0.8 ± 0.3	< 0.001
Brain Stem	D_50%_	38.2 ± 2.3	38.1 ± 2.2	0.4 ± 0.3	0.002
	D_2%_	57.3 ± 6.8	57.1 ± 6.8	0.3 ± 0.2	< 0.001
Parotids	D_50%_	40.9 ± 7.1	41.0 ± 7.0	−0.1 ± 0.6	0.901
	D_2%_	69.3 ± 1.6	69.2 ± 1.6	0.2 ± 0.3	0.136
Lens	D_50%_	4.4 ± 1.9	4.4 ± 1.9	0.7 ± 0.9	0.019
	D_2%_	6.2 ± 2.8	6.2 ± 2.8	0.2 ± 0.6	0.082
Optic nerves	D_50%_	35.9 ± 21.4	35.5 ± 21.5	1.6 ± 4.4	0.097
	D_2%_	54.1 ± 23.7	53.7 ± 23.4	0.4 ± 0.8	0.078
TM-Joints	D_50%_	44.2 ± 6.4	42.0 ± 6.0	5.1 ± 0.7	< 0.001
	D_2%_	67.2 ± 4.3	64.6 ± 4.2	4.5 ± 1.2	< 0.001
Mid-Ears	D_50%_	43.3 ± 4.1	42.4 ± 3.7	2.1 ± 1.7	0.009
	D_2%_	64.2 ± 4.8	62.0 ± 5.0	3.4 ± 1.7	< 0.001
Mandibles	D_50%_	49.5 ± 6.8	46.8 ± 7.2	5.5 ± 1.8	< 0.001
	D_2%_	67.4 ± 4.4	64.2 ± 4.7	4.8 ± 1.5	< 0.001
Temporal lobe	D_50%_	16.8 ± 7.3	16.7 ± 7.3	0.6 ± 0.7	0.003
	D_2%_	64.2 ± 6.0	63.6 ± 6.0	0.9 ± 0.3	< 0.001
Tongue	D_50%_	47.7 ± 6.7	47.4 ± 6.7	0.6 ± 0.3	< 0.001
	D_2%_	65.3 ± 5.3	65.2 ± 5.5	0.2 ± 0.6	0.340

As for the OARs, the D_50%_ increased when D_m_ was converted to D_w_, and this was a statistically significant result except for the optic nerve and parotid gland. The median dose of T-M joints and mandibular in the D_m_ plans were at least 5% less than that in the D_w_ plans. The D_2%_ of spinal cord, brain stem, parotid gland, lens, optic nerves, temporal lobe, and tongue increased by less than 1% from D_m_ to D_w_. However, the D_2%_ of T-M joints and mandibular suffered about 5% change from D_m_ to D_w_.

### D_w_ and D_m_ for lung cancer cases

**Fig. 2:**
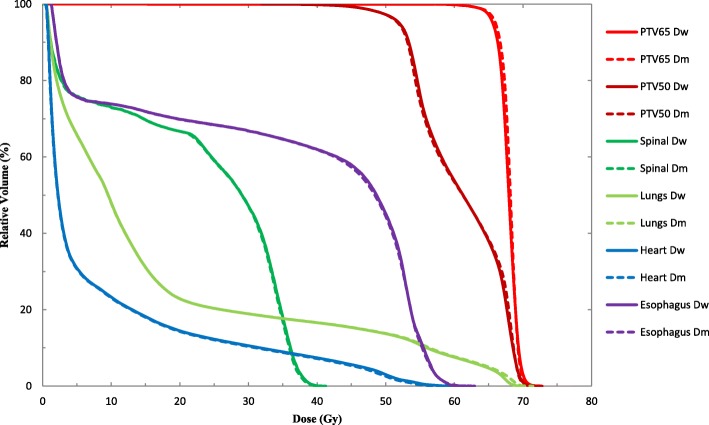
DVH comparison for D_w_ and D_m_ results from the MC-based Monaco TPS for a typical Lung case

Figure 2 shows that, for lung cancer cases, the difference between D_w_ and D_m_ is less obvious than in the NPC cases. Table 2 shows that the D_2%_ of PTV65 and the D_98%_ of PTV50 were statistically significant (*p* <  0.05), and the mean deviation were 0.3 ± 0.4% and − 0.3 ± 0.3%, respectively. There were no other statistically significant differences for other DVH indices evaluated for PTVs. All deviations were with 1%. For the OARs, the median dose D_50%_ of spinal cord and heart were slightly increased from D_m_ to D_w_ with the mean deviation at 0.3 ± 0.3% and 1.1 ± 0.5%, respectively, and this was statistically significant. There were no statistically significant differences between D_w_ and D_m_ in lung and esophagus. For the D_2%_ of spinal cord, lung, esophagus and heart, there were statistically significant differences between D_w_ and D_m_, and the mean deviation were 0.3 ± 0.4%, − 0.6 ± 0.5%, − 0.7 ± 0.5%, and 0.6 ± 0.6%, respectively. All the differences in the DVH indices evaluated were within 2%.

**Table 2:** The mean and standard deviation of D_w_ and D_m_ in dose-volume indices calculated with Monte Carlo for 10 Lung IMRT cases

ROI	Parameter	D_w_(Gy)	D_m_(Gy)	Diff(%)	*p*
PTV65	D_98%_	60.7 ± 2.9	60.6 ± 2.9	−0.2 ± 0.5	0.274
	D_50%_	68.1 ± 0.3	68.3 ± 0.3	−0.3 ± 0.3	0.106
	D_2%_	71.1 ± 0.9	70.9 ± 1.0	0.3 ± 0.4	0.032
PTV50	D_98%_	49.6 ± 1.0	49.8 ± 1.0	−0.3 ± 0.3	0.004
	D_50%_	64.2 ± 4.2	64.2 ± 4.3	−0.1 ± 0.4	0.707
	D_2%_	70.8 ± 1.0	70.6 ± 1.1	0.2 ± 0.4	0.137
Spinal	D_50%_	28.1 ± 9.8	28.1 ± 9.7	0.3 ± 0.3	0.001
	D_2%_	41.2 ± 2.4	41.1 ± 2.4	0.3 ± 0.4	0.046
Lungs	D_50%_	8.5 ± 2.9	8.5 ± 2.9	−0.2 ± 0.2	0.052
	D_2%_	65.8 ± 3.9	66.2 ± 4.1	−0.6 ± 0.5	0.003
Esophagus	D_50%_	40.0 ± 16.9	40.0 ± 16.9	−0.1 ± 0.6	0.718
	D_2%_	60.2 ± 2.9	60.7 ± 3.1	−0.7 ± 0.5	0.004
Heart	D_50%_	6.1 ± 7.0	6.1 ± 7.0	1.1 ± 0.5	0.010
	D_2%_	51.0 ± 10.7	50.5 ± 10.5	0.6 ± 0.6	0.001

### D_w_ and D_m_ for bone target cases

**Fig. 3:**
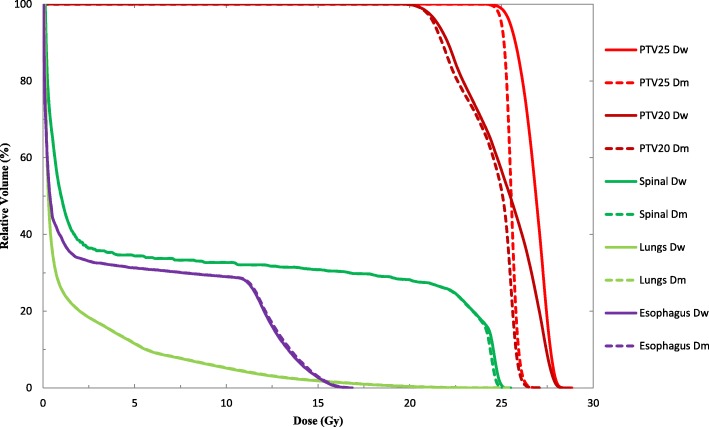
DVH comparison for D_w_ and D_m_ results from the MC-based Monaco TPS for a typical thoracic vertebra metastasis of prostate cancer case

Figure 3 shows that, for bone metastases cases, the differences between D_w_ and D_m_ for PTV targets are more obvious than those in the NPC cases and lung cases. From Table 3, all DVH indices for the PTVs were different with statistical significance (*p* <  0.01). The D_98%_, D_50%_, and D_2%_ deviation of PTV25 were 3.0 ± 1.2%, 3.5 ± 1.4% and 4.4 ± 1.9%, respectively. For the PTV20, D_98%_, D_50%_, and D_2%_ deviations were 2.2 ± 0.7%, 2.8 ± 0.7% and 3.8 ± 1.7%, respectively. There were basically negligible differences between D_w_ and D_m_ in spinal, lung and esophagus. All the differences in the DVH indices evaluated for OARs were within 0.6%.

**Table 3:** The mean and standard deviation of D_w_ and D_m_ in dose-volume indices calculated with Monte Carlo for 10 bone target cases

ROI	Parameter	D_w_(Gy)	D_m_(Gy)	Diff(%)	*p*
PTV25	D_98%_	25.7 ± 0.9	24.9 ± 1.0	3.0 ± 1.2	0.002
	D_50%_	27.2 ± 0.3	26.2 ± 0.4	3.5 ± 1.4	< 0.001
	D_2%_	28.2 ± 0.4	27.0 ± 0.4	4.4 ± 1.9	< 0.001
PTV20	D_98%_	21.6 ± 0.9	21.1 ± 1.0	2.2 ± 0.7	0.019
	D_50%_	25.2 ± 1.7	24.4 ± 1.7	2.8 ± 0.7	< 0.001
	D_2%_	27.9 ± 0.4	26.8 ± 0.3	3.8 ± 1.7	< 0.001
Spinal	D_50%_	14.4 ± 9.9	14.3 ± 9.9	0.4 ± 0.5	0.025
	D_2%_	24.4 ± 1.4	24.3 ± 1.3	0.5 ± 0.3	0.001
Lungs	D_50%_	1.4 ± 1.4	1.4 ± 1.4	0.0 ± 0.3	0.999
	D_2%_	14.2 ± 5.7	14.3 ± 5.7	−0.6 ± 0.6	0.011
Esophagus	D_50%_	5.1 ± 6.6	5.1 ± 6.6	−0.6 ± 1.0	0.950
	D_2%_	21.0 ± 3.6	21.0 ± 3.6	0.1 ± 0.4	0.453

### Dose difference distribution maps

**Fig. 4:**
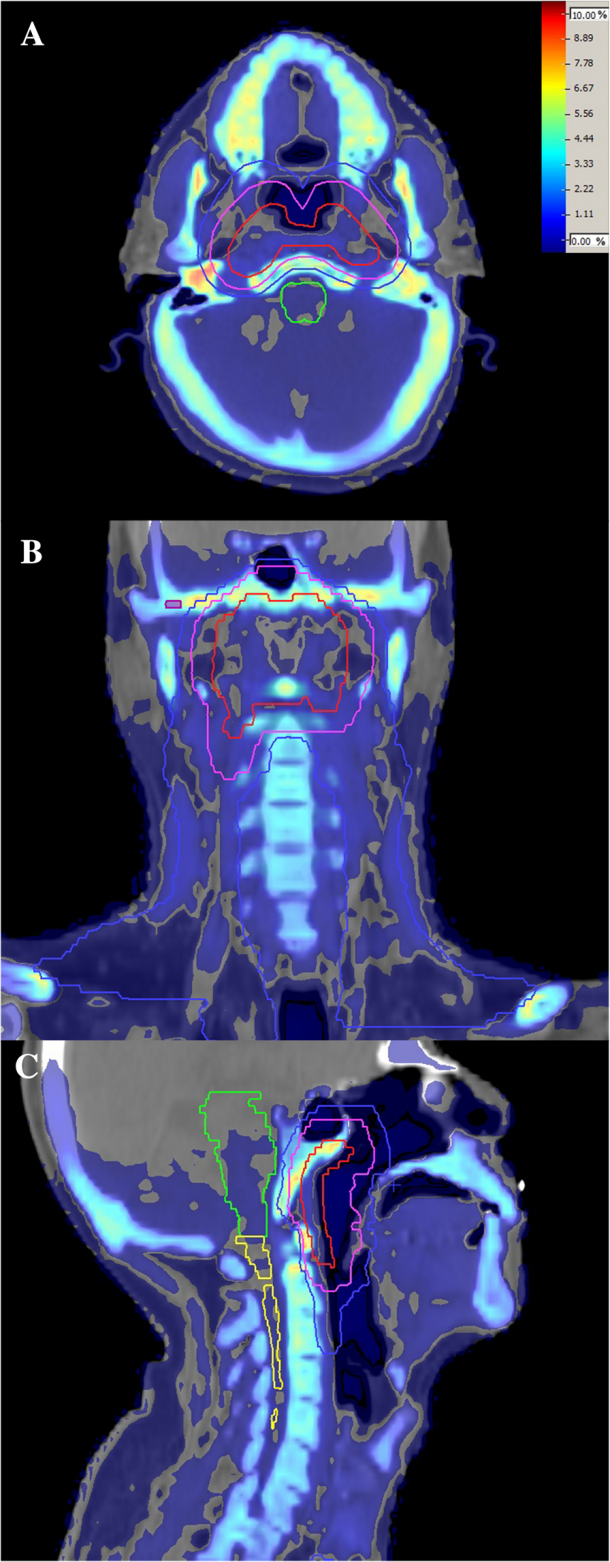
D_w_ and D_m_ dose difference map displayed in axial (**a**), coronal (**b**), and sagittal (**c**) slices in a typical NPC case

**Fig. 5:**
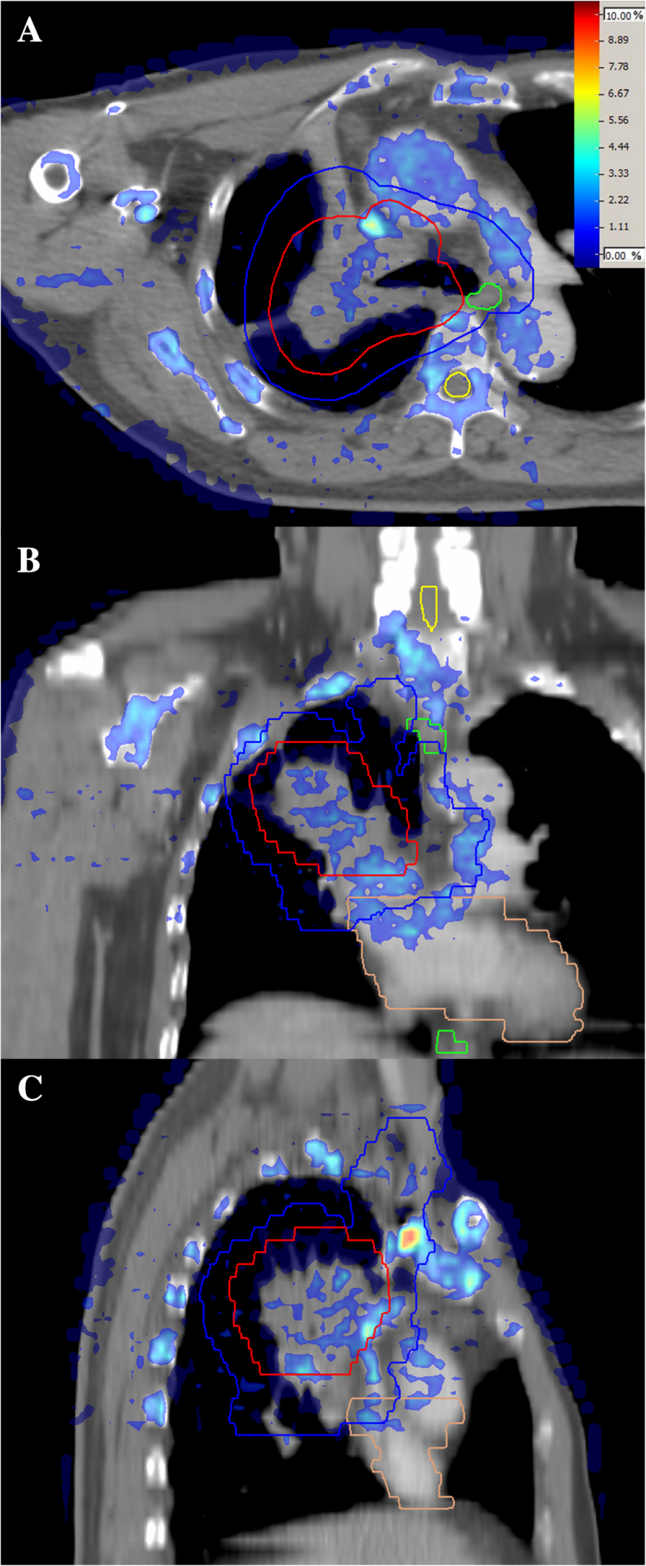
D_w_ and D_m_ dose difference map displayed in axial (**a**), coronal (**b**), and sagittal (**c**) slices in a typical lung case

**Fig. 6:**
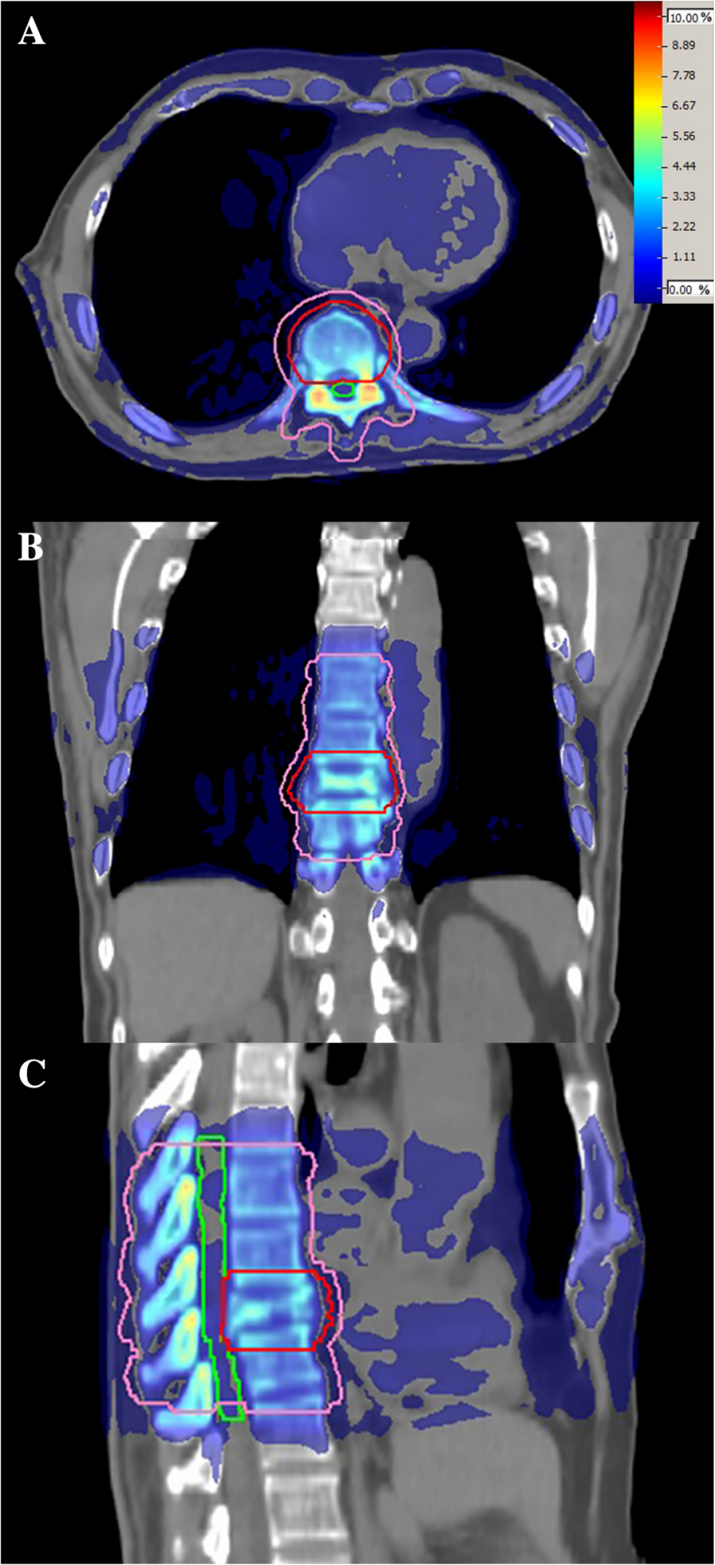
D_w_ and D_m_ dose difference map displayed in axial (**a**), coronal (**b**), and sagittal (**c**) slices in a typical bone target case

By subtracting the re-calculated D_w_ plan and original D_m_ plans, the dose difference of three-dimensional distribution can be obtained. The dose difference (diff) is defined by diff (%) = (D_w_ - D_m_)/ D_p_ × 100, where D_p_ is the prescription dose. Figure 4 shows the difference distribution in three-dimensions of a typical NPC case between D_w_ and D_m_. A typical case of lung cancer is shown in Fig. 5 and a case of bone metastasis is shown in Fig. 6. The blue to purple gradient legend represented the dose difference values ranging from 0 to 10%. It can be seen from Fig. 4 and Fig. 5 that the difference between D_w_ and D_m_ could be higher than 5% in bone, while the differences between D_w_ and D_m_ in soft tissues were less obvious (usually smaller than 3%). From Fig. 6 the differences between D_w_ and D_m_ in thoracic vertebra bone were about 3–8%, a little lower than the result in head bone in Fig. 3. It’s probably because the bone density of the thoracic vertebra is different from that of the head bone.

### Dose verification

At normal QA criterion, 3% dose difference and 3 mm distance to agreement, the gamma pass rates of D_w_ plan and D_m_ plan are all above 94% and very close. But when the tolerances become stricter, the gamma passing rates decreases dramatically, and D_w_ plans gamma pass rates become better than the D_m_ plans (Table 4).

**Table 4:** The local gamma passing percentages at different quality assurance criteria for NPC IMRT cases

Tolerance	Measurement vs D_m_	Measurement vs D_w_	t	*p*
3%&3 mm	94.3 ± 3.2%	97.1 ± 2.2%	−2.464	0.036
2%&2 mm	79.1 ± 2.7%	89.1 ± 1.6%	−2.882	0.018
1%&1 mm	43.6 ± 2.6%	56.1 ± 2.3%	−3.024	0.014

t*, p* values were calculated by paired t-tests using the SPSS 19.0

## Discussions

With the application of MC algorithm for dose calculation in radiation therapy, whether the dose should be calculated to medium or to water has been an unsettled debate [9, 10, 16]. The arguments that support D_w_ include that beam data was measured in water, that the beam output was calibrated in water, and that most clinical experience were based on dose to water, etc. However, the compelling argument to support the use of D_m_ is that it represents the true dose at each location of specific medium. It is the unique advantage of Monte Carlo in that D_m_ can be calculated directly, but D_m_ to D_w_ using stopping power ratios may involve an uncertainty [17]. In reality, different TPS use different dose calculation algorithms to produce D_w_, from direct calculation to applying conversion factors. According to the AAPM TG 105 report [18], when the element components are considered in dose calculation, both D_m_ and D_w_ should be available for evaluation. When comes to a specific clinical situation, the difference between D_m_ and D_w_ should be known. N Dogan et al. [19] showed that converting D_m_ to D_w_ in EGS4 MC-calculated IMRT treatment plans introduces a systematic error in target and critical structure DVHs, and this systematic error may reach up to 5.8% for H&N and 8.0% for prostate cases when the hard-bone-containing structures such as femoral heads are present.

From our work using Monaco for NPC and lung cancer, D_m_ was less than D_w_. The mean deviation for soft tissues was within 2%. For T-M joints and mandibular, the mean deviation was greater than 5%, and in regions of unspecified normal bone the difference could reach 10%. Our results agreed nicely with the work by Siebers et al. [8]. It is interesting to find, based on our study, that there was hardly any difference between D_w_ and D_m_ in low density regions. Although the stopping power ratio for both cortical bone and air can be above 1.10, the stopping power ratio is close to 1 for low density tissues like lung. For this reason, the issue with using D_w_ or D_m_ may have a minimal effect for majority of clinical situations.

The dose difference between D_w_ and D_m_ in bony structures may become clinically significant if the OAR is receiving doses close to its tolerance dose limit which can influence selection or rejection of a particular plan. The dose calculated by MC may need to be carefully evaluated in certain situations, e.g. bone metastasis, bone tumor, or constraining a hot spot in bone that becomes a limiting factor in plan optimization. From the Fig. 3, for PTV of the bone target cases, though the target dose coverages (the target volume (%) received the prescription dose) of D_m_ and converted D_w_ plan were similar, the mean median dose of D_w_ plan increased by 3.5% comparing with that of D_m_ plan (Table 3). That means the dose prescription for bone target could be about 3.5% higher than that of using D_w_ dose, and their treatment response and outcome may need further study in the future.

Previous studies [16, 20] using EGS4/MCSIM Monte Carlo and AXB dose calculations proved that conventional model based algorithms predicted dose distributions in bone that were closer to D_m_ distributions than to D_w_ distributions. It is therefore better to use D_m_ for consistency with previous radiation therapy experience. Our measurements showed that at widely used reference standard, 3% dose difference and 3 mm DTA, the D_m_ and D_w_ plan gamma passing rates were very close, but when the gamma calculation standard became stricter, the D_w_ was closer to the result of measurement than the D_m_. That’s because the MapCheck2 CT images without forcing density were used to calculate the planned dose distribution, where the MapCheck2 detectors are made of high density metallic elements and the detectors are always calibrated by D_w_. The CT scanner used for acquisition of patient simulation images has the limitation of scanning high density material such as the diode and the TPS also has limitation while accepting CT images with high density material. In our practice, D_m_ is used for treatment planning, and physicians and physicists will be consulted in case conversion to D_w_ in bone may affect the decisions to choose the appropriate dose distribution for treatment.

Conversion to D_w_ may be necessary for dose verification in the quality assurance phantom. If a water phantom is used, the difference between D_m_ and D_w_ can be ignored. Kan MW et al. [20] showed that for a heterogeneous phantom with high density materials contained the difference between D_m_ and D_w_ has an effect on the passing rate of QA measurement. Our results (Table 4) showed there were obvious differences between the D_m_ and D_w_ plan gamma passing rates when the QA criteria became strict. A simple method to bypass the problem is to assign a uniform density to the phantom and calculate to either D_m_ or D_w_ in a consistent manner. The choice of an appropriate density needs to be validated by an independent method such as point dose measurement.

## Conclusions

Overall, the dose differences between D_m_ and D_w_ calculated by MC algorithm in Monaco are small in regions that have densities close or low to water. Our results show that dose calculated to medium by Monaco can be used clinically. In high density regions like cortical bone, the difference was 5 to 10%, and this may have a clinical consequence and needs to be carefully considered in certain clinical situations.

## Abbreviations

CTV: Clinical target volume
DD: Dose difference
D_m_: Dose to media
DTA: Distance to agreement
DVH: Dose volume histogram
D_w_: Dose to water
GTV: Gross tumor volume
HU: HOUNSFIELD unit
IMRT: Intensity modulated radiation therapy
MC: Monte carlo
MU: Monitor unit
NPC: Nasopharyngeal carcinoma
OAR: Organ at risk
PTV: Planning target volume
QA: Quality assurance
ROI: Region of interest
TPS: Treatment planning system
